# Waist to hip ratio and trunk to extremity fat (DXA) are better surrogates for IMCL and for visceral fat respectively than for subcutaneous fat in adolescent girls

**DOI:** 10.1186/1743-7075-7-86

**Published:** 2010-12-09

**Authors:** Eray Savgan-Gurol, Miriam Bredella, Melissa Russell, Nara Mendes, Anne Klibanski, Madhusmita Misra

**Affiliations:** 1 Neuroendocrine Unit, Massachusetts General Hospital and Harvard Medical School, Boston, MA, 02114, USA; 2 Pediatric Endocrine Unit, Mass General Hospital for Children and Harvard Medical School, Boston, MA, 02114, USA; 3 Department of Radiology, Massachusetts General Hospital and Harvard Medical School, Boston, MA, 02114, USA

## Abstract

**Background:**

Increased visceral adipose tissue (VAT) and intramyocellular lipids (IMCL) are associated with increased metabolic risk. Clinical and DXA body composition measures that are associated with VAT are generally even more strongly associated with subcutaneous adipose tissue (SAT) reflecting general adiposity, and thus are not specific for VAT. Measures more strongly associated with VAT than SAT (thus more specific for VAT), and predictors of IMCL have not been reported.

**Subjects/Methods:**

We studied 30 girls 12-18 years; 15 obese, 15 normal-weight. The following were assessed: (1) anthropometric measures: waist circumference at the umbilicus and iliac crest (WC-UC and WC-IC), waist-to-hip ratio (WHR), waist-to-height ratio (WHtR), (2) DXA measures: total fat, percent body fat (PBF), percent trunk fat (PTF), trunk-to-extremity fat ratio (TEFR), (3) MRI and 1H-MRS: VAT and SAT (L4-L5), soleus-IMCL.

**Results:**

***Group as a whole: ***WC, trunk fat and PBF were more strongly associated with SAT than VAT; none were specific for VAT. In contrast, PTF and TEFR were more significantly associated with VAT (r = 0.83 and 0.81 respectively, p <0.0001 for both) than SAT (r = 0.77 and 0.75, p < 0.0001 for both). Strongest associations of S-IMCL were with WHR (r = 0.66, p = 0.0004). ***Subgroup analysis: ***In obese girls, WHR and WHtR were more strongly correlated with VAT (r = 0.62 and 0.82, p = 0.04 and 0.001) than SAT (r = 0.41 and 0.73, p not significant and 0.007), and for DXA measures, PTF and TEFR were more significantly associated with VAT (r = 0.70 and 0.72, p = 0.007 and 0.006) than SAT (r = 0.52 and 0.53, p = 0.07 and 0.06). In controls, PTF and TEFR were more strongly correlated with VAT (r = 0.79, p = 0.0004 for both) than SAT (r = 0.71 and 0.72, p = 0.003 for both). WHR was associated with IMCL in obese girls (r = 0.78, p = 0.008), but not controls.

**Conclusion:**

Overall, WHR (anthropometry), and PTF and TEFR (DXA) are good surrogates for IMCL and for visceral fat respectively in adolescent girls.

## Background

Obesity and its morbidities are increasing in prevalence in children [1], and early identification of children at greatest risk for cardiovascular disease and insulin resistance is important to implement preventive and therapeutic strategies. Assessment of fat distribution may be a useful strategy to determine risk for obesity-associated morbidity. Particularly, visceral adipose tissue (VAT) is strongly associated with cardiovascular morbidity and insulin resistance in adults [2,3], and children [4-7]. A relationship has also been demonstrated between intramyocellular lipids (IMCL) and insulin resistance in adults [8,9] and adolescents [8-10]

Approaches to assess fat distribution include anthropometry, bioelectric impedance (BIA), dual-energy x-ray absorptiometry (DXA), computed tomography (CT), magnetic resonance imaging (MRI) and spectroscopy (MRS) [11]. Imaging methods are considered to be the most accurate approaches for *in-vivo *quantification of regional fat [12], and specifically, CT and MRI allow precise quantification of VAT and subcutaneous adipose tissue (SAT). Similarly, MRS can quantify muscle lipid at early stages of insulin resistance and cardiovascular disease. However, quantification of VAT and IMCL using such techniques is expensive, and CT involves radiation exposure, limiting clinical utility. It is important to identify other measures of body composition that are surrogates of VAT and IMCL, and can be utilized as practical clinical tools for detecting those at risk for obesity-related co-morbidities.

Anthropometric measurements such as subscapular and triceps skinfold thickness (SSFT and TSFT), waist circumference at the umbilicus or iliac crest (WC-UC or WC-IC), hip circumference (HC) and waist to hip ratio (WHR) have been used as clinical tools for indirect fat assessment, and increased WC and WHR predict obesity related comorbidities [13,14]. However, studies that assessed anthropometry to predict VAT revealed mixed results in adults, and overall indicated that these measures correlated better with SAT than VAT [12,15], likely because SAT and VAT track together, and individuals with high SAT also have high VAT. Studies have not assessed whether certain measures better predict VAT than SAT.

Importantly, anthropometric predictors of IMCL have not been reported. Whereas IMCL increases with chronic exercise to serve as readily available fuel for muscle contraction in trained athletes, IMCL also increases in obesity from chronic elevation of plasma fatty acids, and reflects a storage form of excess fat [16,17]. In the latter state, IMCL negatively impacts insulin sensitivity. In obese children, IMCL quantification provides an estimate of the chronicity of energy excess and degree of insulin resistance.

DXA is a noninvasive, accurate, reproducible and inexpensive method of measuring regional fat mass [18]. Although DXA cannot differentiate between VAT and SAT, it is unclear whether specific measures assessed by DXA, such as percent trunk fat (PTF) and trunk-to-extremity fat ratio (TEFR), effectively predict VAT or IMCL.

Our objective was to identify anthropometric and DXA measures that best predict VAT (rather than SAT) and IMCL in a homogenous group of normal-weight and obese adolescent girls.

## Subjects and Methods

### Subject Selection

We screened 17 obese and 30 normal-weight controls 12-18 years old for this study. Fifteen qualifying obese subjects were matched for ethnicity, race and bone age (within 2 years) to 15 normal-weight controls. Bone age (a highly reproducible measure of pubertal stage) [19] was used for matching rather than chronological age because obese girls are more pubertally advanced than age-related peers, and body composition in adolescence is determined by pubertal stage rather than chronological age.

Girls were classified as obese if they had a BMI >95^th ^percentile. Normal-weight girls were required to have a BMI between the 15^th^-85^th ^percentiles. Menarchal status did not differ between groups; two normal-weight and three overweight girls were premenarcheal. Subjects were recruited from all ethnic and racial backgrounds through mass mailings to providers, and research listings within the Partners HealthCare network. Each group included 12 Caucasians, two African-Americans, and one subject with multiracial background. The Institutional Review Board of Partners HealthCare system approved the study, and informed assent and consent were obtained.

### Anthropometric measurements

Subjects were weighed to the nearest 0.1 kg in a hospital gown on an electronic scale at our Clinical Research Center. Height was measured to the nearest 0.1 cm on a single stadiometer, and an average of three measurements taken. Abdominal circumference was measured with a plastic tape measure to the nearest 0.1 cm at the level of umbilicus (WC-UC) and iliac crest (WC-IC) at the end of expiration with the subject standing. Waist-to-hip ratio (WHR) was determined (using waist measurements at the level of the umbilicus and maximum hip circumference). Waist-to-height ratio (WHtR) was assessed. Associations of WHR and WHtR with body composition measures were similar when we used WC-UC or WC-IC for these ratios, and we only report data for WHR and WHtR using WC-UC (which provided stronger associations).

### Experimental protocol

Exclusion criteria included pregnancy, use of medications that affect body composition (such as estrogens, progestins, or glucocorticoids), weight loss or gain of >2 kg within 3 months preceding the study, diabetes mellitus, and thyroid disorders. Body composition was determined and ^1^H-MRS performed using a 1.5 T MRI system (Signa 1.5 Tesla; General Electric Medical Systems, Milwaukee, WI) and DXA (Hologic 4500; Waltham, MA). MRI was performed in the fasting state and included measurements of SAT and VAT at L4 using a single axial abdominal MR imaging slice. VAT and SAT were determined based on offline analysis of tracings obtained utilizing commercial software (VITRAK, Merge/eFilm, WI). After an 8-h overnight fast, subjects underwent ^1^H-MRS of calf muscle. A voxel measuring 15 × 15 × 15 mm (3.4 ml) was placed on the axial T1-weighted slice with largest muscle cross-sectional area of the soleus, avoiding visible interstitial tissue, fat, or vessels. Single-voxel ^1^H-MRS data were acquired using point-resolved spatially localized spectroscopy pulse sequence [20]. DXA (Hologic 4500, Waltham, MA) was used to assess total fat, percent body fat (PBF), trunk fat, PTF [(trunk fat/total fat)*100] and TEFR (trunk fat/extremity fat).

### Statistical analysis

JMP Statistical program version 5 (SAS Institute, Cary, NC) was used for analyses. We used Spearman-Rho univariate analysis to detect associations of anthropometric and DXA measures of regional body composition with VAT, SAT and IMCL. Most anthropometric and DXA measures of regional body composition are associated with both VAT and SAT, and these correlations are generally stronger with SAT than VAT, indicating associations with adiposity in general (rather than specifically VAT). We looked for anthropometric and DXA measures in which the correlation coefficients were higher for VAT than SAT as indicative of measures that better differentiated VAT from SAT, as opposed to measures where the correlation coefficients were higher for SAT than VAT, and likely indicated general adiposity. In these exploratory analyses, in addition to requiring the correlation coefficient to be higher for VAT than for SAT, we also arbitrarily required a difference in correlation coefficients of at least 0.05 to be able to differentiate between VAT versus SAT. In addition, we performed quartile analysis and compared the reliability of any two methods for categorizing a specific body composition parameter into four quartiles using kappa statistics. A kappa value of <20 indicates slight agreement, 21-40 fair agreement, 41-60 modest agreement, 61-80 substantial agreement and > 81 almost perfect agreement. Finally, we used stepwise regression to develop predictive equations for VAT, SAT and IMCL, using a p value of 0.10 to enter and leave the model.

## Results

### Clinical Characteristics

Characteristics of the matched subjects are shown in Table 1. The groups did not differ for maturity (bone age or pubertal stage) per study design.

**Table 1:** Demographics of obese and normal-weight girls

	Obese N = 15	Normal-weight N = 15	p
Age (years)	14.0 ± 1.9	15.9 ± 1.7	0.006
Bone age (years)	15.1 ± 1.9	15.8 ± 1.8	ns
Tanner stage (breasts)	4.3 ± 1.0	4.5 ± 1.1	ns
BMI (kg/m^2^)	34.4 ± 7.1	21.7 ± 1.9	< 0.0001
BMI SDS	3.7 ± 1.5	0.1 ± 0.4	< 0.0001
Subscapular skinfold thickness (mm)	23.0 ± 7.7	12.6 ± 4.0	0.0001
Triceps skinfold thickness (mm)	25.9 ± 5.1	17.3 ± 4.2	< 0.0001
Waist circumference (umbilicus) (cm)	105.7 ± 16.8	77.7 ± 5.5	< 0.0001
Waist circumference (iliac crest) (cm)	106.0 ± 16.6	76.0 ± 5.3	< 0.0001
Waist to hip ratio	0.90 ± 0.08	0.79 ± 0.05	0.0007
Waist to height ratio	0.65 ± 0.09	0.48 ± 0.03	< 0.0001
Total lean mass(kg)	53.4 ± 9.1	40.1 ± 4.3	0.0001
Total fat mass (kg)	39.3 ± 13.6	16.5 ± 3.4	0.0001
Percent body fat	40.5 ± 6.0	28.0 ± 4.0	< 0.0001
Trunk fat (kg)	18.0 ± 8.1	6.1 ± 2.1	< 0.0001
Extremity fat (kg)	20.1 ± 6.3	9.5 ± 1.7	< 0.0001
Percent trunk fat (%)	44.6 ± 6.6	36.2 ± 6.0	0.001
Percent extremity fat (%)	52.4 ± 6.1	58.2 ± 5.2	0.009
Trunk to extremity fat ratio	0.88 ± 0.25	0.64 ± 0.11	0.004
Subcutaneous adipose tissue (cm^2^)	449.4 ± 174.9	151.3 ± 54.1	< 0.0001
Visceral adipose tissue (cm^2^)	46.8 ± 18.7	20.8 ± 8.2	0.0003
^1^H-Intramyocellular lipid (soleus) (AU)	13.6 ± 4.7	9.5 ± 5.0	0.04

Means ± S.D.

Ns: not significant; AU: arbitrary units

### Group as a Whole

#### Correlation of Anthropometric and DXA Measures with VAT and SAT

##### Anthropometric Measures

For VAT, strongest associations with anthropometric measures were observed with WC-UC (r = 0.88, p < 0.0001) and WC-IC (r = 0.89, p < 0.0001) (Table 2). Similarly, strongest associations of anthropometric measures with SAT were observed with WC-UC (r = 0.94, p < 0.0001) and WC-IC (r = 0.96, p < 0.0001). Thus, both WC-UC and WC-IC were more strongly associated with SAT than VAT.

**Table 2:** Associations of anthropometric and DXA measures of body composition with MRI and MRS measures in all subjects (n = 30)

	IMCL (AU)		VAT (cm^2^)		SAT (cm^2^)	
	r	p	r	p	r	p
Subscapular skinfold thickness (mm)	0.42	0.04	0.76	< 0.0001	0.80	< 0.0001
Triceps skinfold thickness (mm)	0.34	0.09	0.72	< 0.0001	0.82	< 0.0001
Waist circumference-umbilicus (cm)	0.38	0.06	0.88	< 0.0001	0.94	< 0.0001
Waist circumference-iliac crest (cm)	0.41	0.04	0.89	< 0.0001	0.96	< 0.0001
**Waist to hip ratio**	**0.66**	**0.0004**	0.71	< 0.0001	0.73	< 0.0001
Waist to height ratio	0.41	0.04	0.86	< 0.0001	0.93	< 0.0001
Total fat (kg)	0.46	0.02	0.86	< 0.0001	0.94	< 0.0001
Percent body fat (%)	0.50	0.008	0.87	< 0.0001	0.92	< 0.0001
Trunk fat (kg)	0.44	0.02	0.88	< 0.0001	0.94	< 0.0001
Extremity fat (kg)	0.47	0.01	0.78	< 0.0001	0.90	< 0.0001
Percent extremity fat (%)	-0.33	0.09	-0.78	< 0.0001	-0.70	< 0.0001
**Percent trunk fat (%)**	ns	ns	**0.83**	**< 0.0001**	**0.77**	**< 0.0001**
**Trunk to extremity fat ratio**	ns	ns	**0.81**	**< 0.0001**	**0.75**	**< 0.0001**

ns: not significant

P values <0.10 are reported with corresponding correlation coefficients

IMCL: intramyocellular lipid, VAT: visceral adipose tissue, SAT: subcutaneous adipose tissue

##### DXA Measures

Strongest associations of DXA measures and VAT were observed with trunk fat (r = 0.88, p < 0.0001), PBF (r = 0.87, p < 0.0001), total fat (r = 0.86, p < 0.0001), PTF (r = 0.83, p < 0.0001) and TEFR (r = 0.81, p < 0.0001) (Table 2). Strongest associations of DXA measures and SAT were observed with trunk fat (r = 0.94, p < 0.0001), total fat (r = 0.94, p < 0.0001) and PBF (r = 0.92, p < 0.0001). Associations for TEFR and PTF were stronger for VAT than SAT for the group as a whole.

#### Correlation of Anthropometric and DXA Measures with IMCL

##### Anthropometric Measures

Correlation analyses between anthropometric measurements and IMCL indicated that WHR was the strongest predictor of IMCL (r = 0.66, p = 0.0004).

##### DXA Measures

PBF was the strongest DXA predictor of IMCL (r = 0.50, p = 0.008), although other measures such as total fat, TF and EF were also associated (r = 0.46, 0.44 and 0.47, p = 0.02, 0.02 and 0.01).

### Obese Subjects and Controls

#### Correlation of Anthropometric and DXA Measures with VAT and SAT

##### Anthropometric Measures

Within **obese subjects**, WC-UC and WC-IC showed stronger correlations with SAT (r = 0.82 and 0.89, p = 0.001 and 0.0001) than VAT (r = 0.79 and 0.78, p = 0.002 and 0.003), but neither was specific for VAT (Table 3). However, WHR and WHtR were more strongly correlated with VAT (r = 0.62 and 0.82, p = 0.04 and 0.001) than SAT (0.41 and 0.73, p = not significant and 0.007).

**Table 3:** Associations of anthropometric and DXA measures of body composition with MRI and MRS measures in obese and normal-weight subjects

	Obese girls						Normal-weight girls					
	IMCL (AU)		VAT (cm^2^)		SAT (cm^2^)		IMCL (AU)		VAT (cm^2^)		SAT (cm^2^)	
	r	p	r	p	r	p	r	p	r	p	r	p
Subscapular skinfold thickness (mm)	ns	ns	0.77	0.003	0.95	< 0.0001	ns	ns	ns	ns	0.47	0.09
Triceps skinfold (mm)	ns	ns	0.56	0.06	0.63	0.03	ns	ns	ns	ns	0.53	0.05
Waist circumference-umbilicus (cm)	ns	ns	0.79	0.002	0.82	0.001	ns	ns	0.74	0.002	0.85	< 0.0001
Waist circumference-iliac crest (cm)	ns	ns	0.78	0.003	0.89	0.0001	ns	ns	0.81	0.0005	0.86	< 0.0001
**Waist to hip ratio**	**0.78**	**0.008**	**0.62**	**0.04**	**ns**	**ns**	ns	ns	ns	ns	ns	ns
Waist to height ratio	ns	ns	**0.82**	**0.001**	**0.73**	**0.007**	ns	ns	0.73	0.003	0.86	< 0.0001
Total fat (kg)	ns	ns	0.56	0.05	0.96	< 0.0001	ns	ns	0.70	0.003	0.65	0.009
Percent body fat (%)	0.57	0.05	0.55	0.05	0.77	0.002	ns	ns	0.70	0.003	0.66	0.007
Trunk fat (kg)	0.56	0.06	0.59	0.03	0.91	< 0.0001	ns	ns	0.77	0.0007	0.74	0.002
Extremity fat (kg)	ns	ns	0.54	0.05	0.93	< 0.0001	ns	ns	ns	ns	0.48	0.07
**Percent trunk fat **(%)	0.69	0.01	**0.70**	**0.007**	**0.52**	**0.07**	ns	ns	**0.79**	**0.0004**	**0.71**	**0.003**
Percent extremity fat (%)	-0.59	0.04	-0.70	0.008	-0.53	0.06	ns	ns	-0.80	0.0004	-0.63	0.01
**Trunk to extremity fat ratio**	0.59	0.04	**0.72**	**0.006**	**0.53**	**0.06**	ns	ns	**0.79**	**0.0004**	**0.72**	**0.003**

ns: not significant; P values <0.10 are reported with corresponding correlation coefficients

IMCL: intramyocellular lipid; VAT: visceral adipose tissue; SAT: subcutaneous adipose tissue

In the **normal-weight subgrou**p, similarly, WC-UC and WC-IC showed stronger correlations with SAT (r = 0.85 and 0.86, p < 0.0001 for both) than VAT (r = 0.74 and 0.81, p = 0.002 and 0.0005), but neither was specific for VAT (Table 3). WHtR (but not WHR) was associated with both SAT and VAT, however, associations were again stronger for SAT (r = 0.86, p < 0.0001) than VAT (r = 0.73, p = 0.003).

##### DXA Measures

PTF had stronger correlations with VAT in both groups (r = 0.70, p = 0.007 in obese; r = 0.79, p = 0.0004 in normal-weight) compared with SAT (r = 0.52, p = 0.07 in obese; r = 0.71, p = 0.003 in normal-weight) (Table 3). Similarly, TEFR had stronger correlations with VAT in both groups (r = 0.72, p = 0.006 in obese; r = 0.79, p = 0.0004 in normal-weight) compared with SAT (r = 0.53, p = 0.06 in obese; r = 0.72, p = 0.003 in normal-weight). Thus, PTF and TEFR better differentiated VAT from SAT in both groups. Other DXA measures such as total fat, PBF and trunk fat behaved differently in obese versus normal weight girls. They better predicted SAT (r≥0.77, p ≤ 0.002) than VAT (r ≥ 0.54, p ≤ 0.05) in obese girls, and VAT (r≥0.70, p ≤ 0.003) rather than SAT (r ≥ 0.65, p ≤ 0.009) in controls.

#### Correlation of Anthropometric and DXA Measures with IMCL

##### Anthropometric Measures

Subgroup analysis showed that WHR was strongly associated with IMCL in obese girls (r = 0.78, p = 0.008), but not controls (r = 0.36, p = 0.16) (Table 3).

##### DXA Measures

PTF and TEFR was positively associated with IMCL in obese girls, (r = 0.69 and 0.59, p = 0.01 and 0.04) (Table 3). No significant associations were noted of DXA measures with IMCL in controls.

### Quartile Analysis and Degree of Agreement

We next performed quartile analysis to determine the degree of agreement [kappa (κ) statistics] for these associations. Using this method, the anthropometric and DXA measures with greatest agreement with VAT quartiles were WC-U (κ = 0.64) and total fat (κ = 0.62) quartiles respectively. Associations were generally similar within obese girls. The anthropometric and DXA measures with greatest agreement for predicting SAT quartiles were WC-IC (κ = 0.79) and PBF (κ = 0.71) quartiles respectively. Strongest associations of S-IMCL were with WHR (r = 0.66, p = 0.0004) and PBF (r = 0.50, p = 0.008). The variable with greatest agreement with S-IMCL was WHR (κ = 0.50).

Of note, WC-IC, WC-U, trunk fat and PBF were strongly associated with both VAT and SAT. Associations were generally stronger for SAT than VAT, and no measurement was specific for SAT or VAT. In contrast, PTF and TEFR measured by DXA were more significantly associated with VAT (r = 0.83 and 0.81, p < 0.0001 for both) than SAT (r = 0.77 and 0.75, p < 0.0001). Kappa statistics for agreement between PTF and TEFR quartiles with VAT quartiles was 0.38 (for both), compared with 0.19 with SAT quartiles.

### Regression Modeling (Table 4)

**Table 4:** Regression modeling to determine independent anthropometric and DXA predictors of MRI measures of body composition (all subjects; n = 30)

Anthropometric Predictors									
	**Visceral** **Fat** **(cm^2^)**			**Subcutaneous** **Fat** **(cm^2^)**			**Intramyocellular** **Lipid** **(AU)**		
	**P**	**r^2^**	**Total r^2^**	**p**	**r^2^**	**Total r^2^**	**p**	**r^2^**	**Total r^2^**

BMI-SDS	0.002	0.71	**0.77**	< 0.0001	0.90	0.90	-	-	
WHR	0.02	0.06		-	-		0.002	0.35	0.35
									
Bone age	-	-		0.02	0.02		0.0009	0.44	**0.62**
BMI-SDS	0.002	0.71	**0.77**	< 0.0001	0.90	**0.93**	-	-	
WHR	0.02	0.06		0.06	0.01		0.004	0.18	

**DXA Predictors**									

	**Visceral** **Fat** **(cm^2^)**			**Subcutaneous** **Fat** **(cm^2^)**			**Intramyocellular** **Lipid** **(AU)**		
	**p**	**r^2^**	**Total r^2^**	**p**	**r^2^**	**Total r^2^**	**p**	**r^2^**	**Total r^2^**

Total fat (kg)	0.003	0.60	0.72	< 0.0001	0.96	**0.97**	0.06	0.14	0.14
PTF	0.003	0.12		0.04	0.006		-	-	

Total fat (kg)	0.0007	0.60	0.73	< 0.0001	0.96	**0.97**	0.06	0.14	0.14
TEFR	0.003	0.13		0.03	0.007		-	-	

Bone age	0.02	0.06	**0.78**	-	-		0.0009	0.29	**0.46**
Total fat (kg)	0.0006	0.60		< 0.0001	0.96	**0.97**	0.01	0.17	
PTF	0.002	0.12		0.04	0.006		-	-	

Bone age	0.03	0.05		-	-		0.0009	0.29	**0.46**
Total fat (kg)	0.0002	0.60	**0.78**	< 0.0001	0.96	**0.97**	0.01	0.17	
TEFR	0.003	0.13		0.03	0.007		-	-	

BMI-SDS: body mass index standard deviation scores; WHR: waist-to-hip ratio, PTF: percent trunk fat; TEFR: trunk-to-extremity fat ratio

We then performed stepwise regression analysis to determine predictive models for VAT, SAT and IMCL. The first model (two independent variables) included a measure of total body adiposity (BMI-SDS for anthropometric predictors, total fat for DXA predictors) and a measure of regional adiposity (WHR for anthropometric predictors, PTF or TEFR for DXA predictors). The second model (three independent variables) also included bone age to control for maturity. The best predictors of VAT, SAT and S-IMCL are indicated in Table 4.

Amongst anthropometric predictors, the strongest were (i) BMI-SDS and WHR for VAT (77% of variability explained), (ii) BMI-SDS, WHR and bone age for SAT (93% of variability), and (iii) bone age and WHR for S-IMCL (62% of variability). WHR accounted for 6% and 18% of the variability in VAT and S-IMCL, but only 1% of the variability of SAT after controlling for BMI-SDS and bone age. The predictive models were as follows:

$$\begin{array}{l} \text{VAT}\left({\text{cm}}^{\text{2}}\right)=-\text{42}.\text{7}+\text{5}.\text{5}*\text{BMI}-\text{SDS}+\text{79}.\text{7}*\text{WHR}\\ \text{SAT}\left({\text{cm}}^{\text{2}}\right)=-\text{433}.\text{3}+\text{16}.\text{5}*\text{bone age}+\text{81}.\text{9}*\text{BMI}-\text{SDS}+\text{397}.\text{6}*\text{WHR}\\ \text{IMCL }\left(\text{AU}\right)=\text{12}.\text{1 }-\text{1}.\text{7}*\text{bone age}+\text{29}.\text{7}*\text{WHR} \end{array}$$

For DXA predictors, we replaced BMI-SDS with total fat as the latter was a stronger predictor of all regional fat measures than BMI-SDS. We used total fat rather than PBF because of higher predictive values when total fat was used in these models. The strongest DXA predictors were (i) total fat, bone age and TEFR (or PTF) for VAT (78% of variability explained), (ii) total fat and TEFR (or PTF) for SAT (97% of variability), and (iii) bone age and total fat for S-IMCL (46% of variability). PTF and TEFR accounted for 12-13% of the variability of VAT as opposed to only 0.6-0.7% of the variability in SAT, and did not predict S-IMCL in these regression models. The predictive models were as follows:

$$\begin{array}{c} \text{VAT}\left({\text{cm}}^{\text{2}}\right)=\text{21}.0-\text{2}.\text{4}*\text{bone age}+0.\text{8}*\text{total fat}\left(\text{kg}\right)+\text{39}.0*\text{TEFR},\text{or}\\ \;=\text{5}.\text{1}-\text{2}.\text{6}*\text{boneage}+0.\text{7}*\text{total fat}+\text{1}.\text{2}*\text{PTF}\\ \text{SAT}\left({\text{cm}}^{\text{2}}\right)\;=-\text{124}.\text{8}+\text{13}.0*\text{total fat}\left(\text{kg}\right)+\text{1}00.\text{5}*\text{TEFR},\text{or}\\ =-\text{166}.\text{1}+\text{12}.\text{9}*\text{total fat}\left(\text{kg}\right)+\text{2}.\text{9}*\text{PTF}\\ \text{IMCL}\left(\text{AU}\right)=\text{32}.0\text{4}-\text{1}.\text{61}*\text{bone age}+0.\text{16}*\text{total fat}\left(\text{kg}\right) \end{array}$$

## Discussion

MRI and CT currently provide the best estimates of VAT and IMCL, however, the cost of both modalities and radiation exposure with CT limit their use as screening tools for obesity-related morbidities. Our study shows that specific anthropometric and DXA measurements may serve as cost-effective clinical surrogates for VAT and IMCL. Unlike other measures, PTF and TEFR are better associated with VAT than SAT, and may be reasonable surrogates of VAT in female adolescents. Similarly, these are the first data we are aware of that show that WHR is a good surrogate for IMCL.

Studies in adults have shown variable results in the correlation of anthropometric measures with VAT [12,21-23]. One study [12] compared BIA and anthropometric data with MRI measures of VAT, and reported that WHR was associated with VAT. In contrast, another study [22] showed that WC was more strongly associated with VAT than was WHR and that DXA did not offer any advantage over anthropometry for VAT estimation. However, this did not address the issue that these anthropometric measures are associated with both VAT and SAT, and generally more strongly with SAT than VAT.

In children, fat distribution is influenced by gender, ethnicity and puberty. Brambilla *et al*. [6] cross validated anthropometry against MRI for assessment of VAT in children by pooling data from seven investigators. They identified WC as the best predictor of VAT in both normal-weight and obese subjects. However, predictors of VAT strongly predicted SAT, and the study again did not determine which anthropometric measure best differentiated VAT versus SAT. In our study, we examined normal-weight and obese girls and show that anthropometric measures, including WC and WHR, are associated with both VAT and SAT and none are specific for VAT. These findings are consistent with those from previous studies [6,12]. However, in subgroup analysis, WHR was more strongly associated with VAT than SAT in obese girls, indicating that WHR may be a reasonable surrogate for VAT in obese girls at office visits.

We then evaluated DXA measures as possible surrogates for VAT, and found that total fat, PBF and TF correlated more strongly with SAT than VAT. However, unlike other measures, PTF and TEFR were more strongly associated with VAT than SAT in obese and normal-weight subjects (validated by kappa statistics). Therefore, our data suggest that specific DXA measures may provide clinically useful data for adolescents at risk of obesity-related comorbidities by better differentiating VAT from SAT. This is particularly important given that VAT, not SAT, determines insulin resistance and other morbidities. Additionally, we provide predictive equations for VAT using anthropometric and DXA measures.

IMCL, detected by MRS, is an important determinant of insulin resistance in children and adults [8,9,17]. In conditioned athletes, IMCL increases and serves as a source of reserve fuel for times of exercise. However, in obese individuals, IMCL increases consequent to increased dietary fat and circulating fatty acids. Thus, IMCL is an excellent indicator of energy excess, and predicts insulin resistance. The mechanism whereby IMCL induces insulin resistance is unclear, but may be associated with diacylglycerol (DAG) and ceramide accumulation, lipid metabolites known to inhibit insulin signaling, in myotubes. *In vitro *studies indicate that exposure to saturated fatty acids increases DAG and ceramide in myotubes, and inhibits insulin stimulated glycogen synthesis and activation of Akt/protein Kinase B (obligate intermediate in pathway of anabolic metabolism) [24,25]. Similarly, consumption of a diet rich in saturated fats increases DAG in myotubes in rodents, with decreased insulin-stimulated glucose uptake [26]. There are no data that examine whether anthropometric indices can serve as surrogates for IMCL in obese children. We evaluated the usefulness of anthropometry and DXA in predicting IMCL and showed that WHR was the anthropometric measure most strongly associated with IMCL. Of note, WHR was associated with IMCL in obese, but not normal-weight girls.

Limitations of our study include the sample size and the single gender study. This limits the generalizability as fat compartments and anthropometric measures vary between genders consequent to hormonal and physiological differences. However, limiting our subjects to adolescent girls allows for homogeneity of the study group, and provides useful preliminary data to be confirmed in larger studies.

## Conclusion

In summary, our data indicate that although anthropometric measures are associated with VAT and SAT, none are specific for VAT for the group as a whole, and DXA appears to offer an advantage over anthropometry for estimating VAT. Overall, for the group as a whole, WHR was a good surrogate for IMCL, while PTF and TEFR best differentiated VAT from SAT. In obese girls, WHR was again a good surrogate for IMCL, while WHR, WHtR, PTF and TEFR differentiated VAT from SAT. In normal-weight controls, no anthropometric or DXA measure was a good predictor of IMCL, but PTF and TEFR were good surrogates for VAT and best differentiated VAT from SAT. If replicated in both genders and a larger population, specific DXA measures, such as PTF and TEFR, may be utilized to screen adolescents at higher risk for obesity-associated morbidities based on their ability to better determine VAT than SAT, particularly in a research setting. Importantly, the amount of radiation exposure from whole body DXA is less than 3 microSieverts for adolescents and adults, and an annual radiation dose of <10 microSieverts is considered a negligible individual dose by the National Council of Radiation Protection and Measurements (NCRP) [27]. In a clinical setting, WHR and WHtR can be effectively used to predict regional body composition in obese girls.

## Abbreviations

VAT: visceral adipose tissue; IMCL: intramyocellular lipids; BIA: bioelectric impedance; DXA: dual-energy x-ray absorptiometry; CT: computed tomography; MRI: magnetic resonance imaging; MRS: MR spectroscopy; SAT: subcutaneous adipose tissue; SSFT: subscapular skinfold thickness; TSFT: triceps skinfold thickness; WC-UC: waist circumference at the umbilicus; WC-IC: waist circumference at the iliac crest; HC: hip circumference; WHR: waist to hip ratio; PTF: percent trunk fat; TEFR: trunk-to-extremity fat ratio; BMI: body mass index; WHtR: waist to height ratio; PBF: percent body fat

## Competing interests

The authors have no financial or non-financial competing interests to declare.

## Authors' contributions

ESG and MR worked on data analysis and the writing of the manuscript. MB assisted with study design, image acquisition using MRI and MRS techniques and the writing of the manuscript. NM worked on study conduct, data handling and data analysis. AK and MM worked on study design, study conduct, data analysis and the writing of the manuscript. All authors read and approved the final manuscript.
